# *APC* Splicing Mutations Leading to In-Frame Exon 12 or Exon 13 Skipping Are Rare Events in FAP Pathogenesis and Define the Clinical Outcome

**DOI:** 10.3390/genes12030353

**Published:** 2021-02-28

**Authors:** Vittoria Disciglio, Giovanna Forte, Candida Fasano, Paola Sanese, Martina Lepore Signorile, Katia De Marco, Valentina Grossi, Filomena Cariola, Cristiano Simone

**Affiliations:** 1 Medical Genetics, National Institute of Gastroenterology “S. de Bellis” Research Hospital, Castellana Grotte, 70013 Bari, Italy; forte.labsimone@gmail.com (G.F.); fasano.labsimone@gmail.com (C.F.); sanese.labsimone@gmail.com (P.S.); leporesignorile.labsimone@gmail.com (M.L.S.); demarco.labsimone@gmail.com (K.D.M.); grossi.labsimone@gmail.com (V.G.); filo.cariola@irccsdebellis.it (F.C.); 2 Department of Biomedical Sciences and Human Oncology (DIMO), Medical Genetics, University of Bari Aldo Moro, 70124 Bari, Italy

**Keywords:** familial adenomatous polyposis, *APC*, splicing, exon skipping, FAP pathogenesis

## Abstract

Familial adenomatous polyposis (FAP) is caused by germline mutations in the tumor suppressor gene *APC*. To date, nearly 2000 *APC* mutations have been described in FAP, most of which are predicted to result in truncated protein products. Mutations leading to aberrant *APC* splicing have rarely been reported. Here, we characterized a novel germline heterozygous splice donor site mutation in *APC* exon 12 (NM_000038.5: c.1621_1626+7del) leading to exon 12 skipping in an Italian family with the attenuated FAP (AFAP) phenotype. Moreover, we performed a literature meta-analysis of *APC* splicing mutations. We found that 119 unique *APC* splicing mutations, including the one described here, have been reported in FAP patients, 69 of which have been characterized at the mRNA level. Among these, only a small proportion (9/69) results in an in-frame protein, with four mutations causing skipping of exon 12 or 13 with loss of armadillo repeat 2 (ARM2) and 3 (ARM3), and five mutations leading to skipping of exon 5, 7, 8, or (partially) 9 with loss of regions not encompassing known functional domains. The *APC* splicing mutations causing skipping of exon 12 or 13 considered in this study cluster with the AFAP phenotype and reveal a potential molecular mechanism of pathogenesis in FAP disease.

## 1. Introduction

Familial adenomatous polyposis (FAP; OMIM # 175100) is an autosomal dominant disorder characterized by the development of hundreds to thousands of colorectal adenomatous polyps, which, if left untreated, progress to colorectal cancer (CRC) [1,2,3]. FAP patients may develop various extracolonic manifestations, including desmoid tumors, gastrointestinal polyposis, hepatoblastoma, thyroid cancer, and other malignancies [4,5,6,7,8]. Based on the number of colorectal polyps, onset age, and extracolonic manifestations, FAP can be classified into four forms: (1) classic FAP with profuse polyposis (>1000 adenomas); (2) classic FAP with intermediate colonic polyposis (100–1000 adenomas); (3) attenuated FAP (AFAP, <100 adenomas); (4) gastric polyposis and desmoid FAP (GD-FAP), which is characterized by less than 50 polyps, a higher risk of developing desmoid tumors, and a greater susceptibility to give rise to profuse gastric polyposis or adenomas [4,9]. 

FAP is caused by germline mutations in the tumor suppressor gene *APC*, which is located on chromosome 5 and comprises 15 translated exons. Depending on the tissues in which it is expressed, *APC* undergoes alternative splicing leading to multiple protein variants whose molecular weight varies from 90 to 300 kDa [10,11,12,13,14,15,16,17]. The most abundant *APC* transcript encodes a 2843 amino acid protein [18]. 

To date, nearly 2000 *APC* mutations have been described in FAP. The vast majority of these mutations are predicted to result in truncated protein products due to nonsense or frameshift variants or large genomic deletions [19]. Mutations predicted to result in *APC* aberrant splicing have rarely been reported. Isoforms lacking exon 9 or exon 14 owing to splice site mutations have also been associated with FAP disease [20,21,22,23]. 

In this study, we identified and molecularly characterized a novel germline heterozygous splice donor site mutation in *APC* exon 12 (NM_000038.5: c.1621_1626+7del) segregating with the AFAP phenotype in an Italian family. Moreover, we expanded our investigation by performing a meta-analysis to correlate all molecularly characterized *APC* exon 12 and exon 13 splicing mutations with FAP clinical phenotypes.

## 2. Materials and Methods

### 2.1. Patient Recruitment 

The index patient underwent genetic testing following informed consent. Molecular testing carried out in this study is based on the routine clinical diagnostic assessment performed at our Institute. Written informed consent to perform genetic testing and further studies was obtained from the patient using a form approved by the competent ethics committee, in line with the principles of the Declaration of Helsinki and any other applicable local ethical and legal requirements (protocol code N° 170-date of approval 31 October 2016).

### 2.2. Mutation Analysis 

Genomic DNA was extracted from peripheral blood with the QIAamp DNA Blood Mini Kit (Qiagen, Carlsbad, CA, USA) according to the manufacturer’s instructions. *APC* complete coding region was screened for mutations as previously described [24] using primer sequences previously published by Groden J et al. [12]. In order to scan the exons for mutations, the *APC* gene was divided into 23 amplicons using specific primer pairs, with seven amplicons covering exon 15. Each amplicon was sequenced in forward and reverse directions with the same primers used for PCR amplification or internal sequencing primers (Table S1). PCR sequencing and capillary electrophoresis were performed on an Applied Biosystems 3130 Genetic Analyzer (Thermo Fisher Scientific, Waltham, MA, USA). Mutations and polymorphisms were confirmed in independently amplified PCR products. The global population frequency of the identified *APC* variant was retrieved from the 1000 Genome [25,26], dbSNP [27,28], gnomAD [29,30], and NHLBI Exome Sequencing Project (ESP) [31,32] databases. Moreover, the HGMD Professional [33], InSiGHT [34,35], and Clinvar [36,37] databases were interrogated to assess the pathogenicity of the identified variant.

To evaluate the effect of the *APC* c.1621_1626+7del mutation on RNA splicing, four splice site prediction algorithms integrated into Alamut Visual version 2.15 (Sophia Genetics SAS; Bidart, France) were interrogated simultaneously: Splice Site Finder (SSF), MaxEntScan (MES), Splice Site Prediction by Neural Network (NNS), and Gene Splicer (GS). The default thresholds of each tool were used for the analysis. A variation of more than 10% in at least two algorithms was considered as having an effect on the splicing process. 

The identified variant was classified according to the American College of Medical Genetics and Genomics (ACMG) and the Association of Molecular Pathology (AMP) variant classification scheme [38]. 

### 2.3. RT-PCR and mRNA Analysis

Total RNA from peripheral blood was extracted with the QIAamp RNA Blood Mini Kit (Qiagen) according to the manufacturer’s instructions. One microgram of RNA was reverse-transcribed to cDNA using the Maxima H Minus First Strand cDNA Synthesis Kit (Thermo Fisher Scientific). The 5′ and 3′ flanking regions of the *APC* mutation site (NM_000038.5: c.1621_1626+7del) were amplified using the Phusion Hot Start II DNA Polymerase (Thermo Fisher Scientific) and the following primers (10 pmol each): APC_Ex10-11_Fw (NM_000038.5) GAATGAACTAGGGGGACTACAGGC, and APC_Ex13-14_Rv (NM_000038.5) GGGTTGATTCCTTTTTAACTTC. PCR amplification was carried out at 98 °C for 30 s, followed by 35 cycles at 98 °C for 10 s, 60 °C for 10 s, and 72 °C for 15 s, with a final elongation at 72 °C for 5 min. PCR products were loaded onto 3% agarose gel in 0.5X TBE and visualized using SYBR Safe DNA Gel Stain (Thermo Fisher Scientific). Sequencing and capillary electrophoresis were performed on an Applied Biosystems 3130 Genetic Analyzer. 

### 2.4. Cell Line

The HEK-293 cell line was purchased from ATCC and cultured in DMEM high glucose (HG), without pyruvate (Thermo Fisher Scientific) with 10% FBS (Thermo Fisher Scientific), 1% pyruvate (Thermo Fisher Scientific), 1% NEAA (Thermo Fisher Scientific), and 100 U/ml penicillin–streptomycin (Thermo Fisher Scientific) in a 37 °C and 5% CO_2_ incubator. The cell line was tested to be mycoplasma-free according to Venor®GeM Advance kit (Minerva Biolabs, Berlin, Germany) at multiple times throughout the study. 

### 2.5. Plasmid Construct and Expression

Fragments with the wild type or mutant alleles containing *APC* exon 12 (NM_000038.5), flanked by upstream (311 nt) and downstream (447 nt) intronic sequences, were amplified using the following primers: Cloning_APC_Fw_EcoRI ACCAGTGAATTCGACCAAGGCAAGTGTTACACAC, and Cloning_APC_Rv_BamHI ACCGATGGATCCTCCTAAATGCTACTACAGTGCC. Fragments were cloned into the splicing vector pSPL3, linearized with EcoRI and BamHI. All constructs were confirmed by direct sequencing.

### 2.6. In Vitro Splicing Assay

HEK-293 cells were transfected using Lipofectamine 3000 (Thermo Fisher Scientific) according to the manufacturer’s instruction for 24 h. Cells were harvested, total RNA was extracted with the PureLink™ RNA Mini Kit (Thermo Fisher Scientific) according to the manufacturer’s instructions, and used for RT-PCR to confirm splicing patterns. cDNA was synthesized as described above and used as a template for PCR amplification with the following vector-specific primers: SD6_ FW GTCTGAGTCACCTGGACAACC and SA2_ RV GATCTCAGTGGTATTTGTGAGC. PCR amplification was carried out with the Phusion High-Fidelity DNA Polymerase at 98 °C for 30 s, followed by 35 cycles at 98 °C for 10 s, 52 °C for 10 s, and 72 °C for 15 s, with a final elongation at 72 °C for 5 min. PCR products were loaded onto 2% agarose gel in 0.5X TBE and visualized using SYBR Safe DNA Gel Stain. Sequencing and capillary electrophoresis were performed on an Applied Biosystems 3130 Genetic Analyzer. 

### 2.7. Meta-Analysis

The meta-analysis of *APC* splicing mutations was performed on the Human Gene Mutation Database Professional (HGMD Professional; Qiagen), a comprehensive collection of germline mutations in nuclear genes that are associated with human-inherited diseases [33]. We reviewed all the papers identified in the aforementioned database and collected relevant clinical information (i.e., gender, age at diagnosis, gastric or colonic polyposis, and specific *APC* mutations). Studies including patients without clinical information were excluded. 

## 3. Results

### 3.1. Clinical History and Genetic Findings

**Figure 1:**
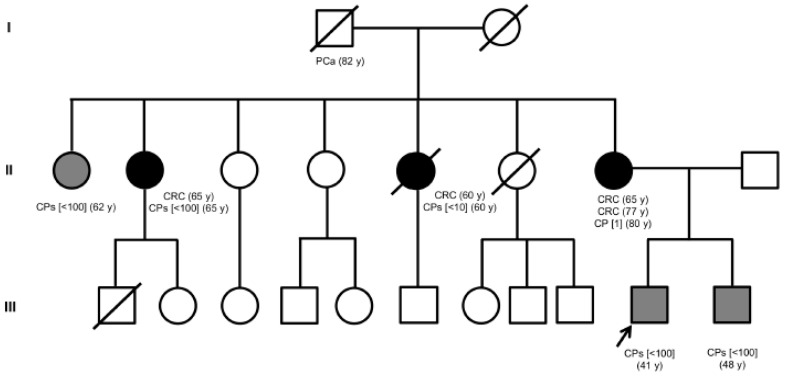
Pedigree of the Italian family involved in this study. Squares indicate men, circles represent women. The arrow indicates the index case. Unfilled symbols indicate unaffected individuals. Slashed symbols denote dead individuals. Black-filled symbols denote individuals with colorectal cancer and polyposis, while gray-filled symbols correspond to patients with colorectal polyposis. The following information is given below each filled symbol: clinical manifestations (CRC = colorectal cancer; CPs = colon polyps, with the number of polyps indicated in square brackets; Pca = prostate cancer), age at diagnosis (y = years).

The index case was a 53-year-old male referred to our institution for genetic counseling. The patient presented at 41 years of age with an attenuated colorectal phenotype (AFAP phenotype), which is consistent with the presence of two intestinal polyps. Histological examination revealed tubular adenomas measuring 4 and 6 mm with moderate-grade dysplasia. After one year, colonoscopy examination did not show evidence of polyposis. The patient was followed up with yearly colonoscopy, which showed three tubular adenomas (2.5 mm) with moderate-grade dysplasia in the large bowel at the age of 43 and two adenomatous polyps (2 mm) with high-grade dysplasia in the sigmoid colon at the age of 44. The patient developed small polyps at the age of 48 and numerous sessile polyps throughout the colon at the age of 51. After one year, colonoscopy examination revealed the presence of various polypoid and non-polypoid lesions in the transverse and descending colon. The patient had a positive family history of colorectal cancer and colon polyposis (Figure 1). 

**Figure 2:**
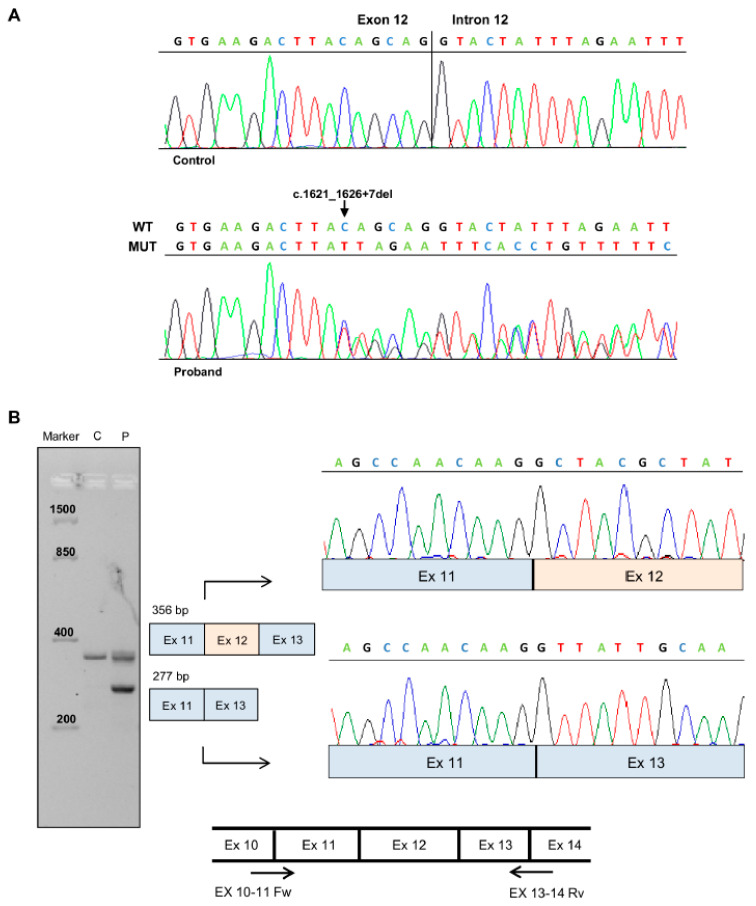
Characterization of *APC* splicing mutation c.1626_1627+7del. (**A**) Sequencing electropherograms of genomic DNA from a healthy control individual and the proband, showing the splicing mutation c.1626_1627+7del. (**B**) Left: Agarose gel electrophoresis showing the RT-PCR analysis of mRNA extracted from peripheral blood of the patient (P) carrying the *APC* c.1621_1626+7del mutation and a healthy control (C). Amplified products obtained with primers spanning *APC* exon 10–11 and 13–14 boundaries were separated on 3% agarose gel and independently sequenced. Center: Schematic diagrams showing the wild type amplification product (356 bp) and the altered-splicing amplification product lacking *APC* exon 12 (277 bp). Right: Sequencing electropherograms of cDNA splicing isoforms generated from the wild type and mutant RT-PCR products. Bottom: Diagram showing the localization of the primers (indicated as arrows) used for RT-PCR experiments.

The brother of the proband exhibited signs of attenuated FAP, showing diffuse polyposis of the colon (<100), and underwent subtotal colectomy at 48 years of age. The mother of the proband developed colorectal carcinomas at the age of 65 and 77. Moreover, she developed an adenomatous polyp at the age of 80. Evaluation of the family history revealed that a maternal aunt of the proband underwent total colectomy for multiple colon polyps (<100). Furthermore, two maternal aunts developed adenomatous polyps of the colon and were diagnosed with colorectal cancer at the age of 60 and 65, respectively (Figure 1). The *APC* mutation analysis performed on the proband’s DNA revealed a heterozygous splicing mutation in *APC* exon 12 (NM_000038.5: c.1621_1626+7del) (Figure 2A).

The identified variant was found to be rare since it was not listed in global population databases (1000 Genome, dbSNP, gnomAD, NHLBI ESP). Moreover, this variant has never been reported in major disease-associated databases (HGMD Professional, InSiGHT, and Clinvar). *In silico* analysis using four algorithms (SSF, MES, NNS, and GS) integrated into Alamut Visual version 2.15 (Sophia Genetics SAS) revealed that *APC* c.1621_1626+7del mutation may result in a splice defect due to loss of the canonical donor site at position c.1626 of the *APC* gene (Figure S1). 

### 3.2. Analysis of Patient’s Processed Transcripts

**Figure 3:**
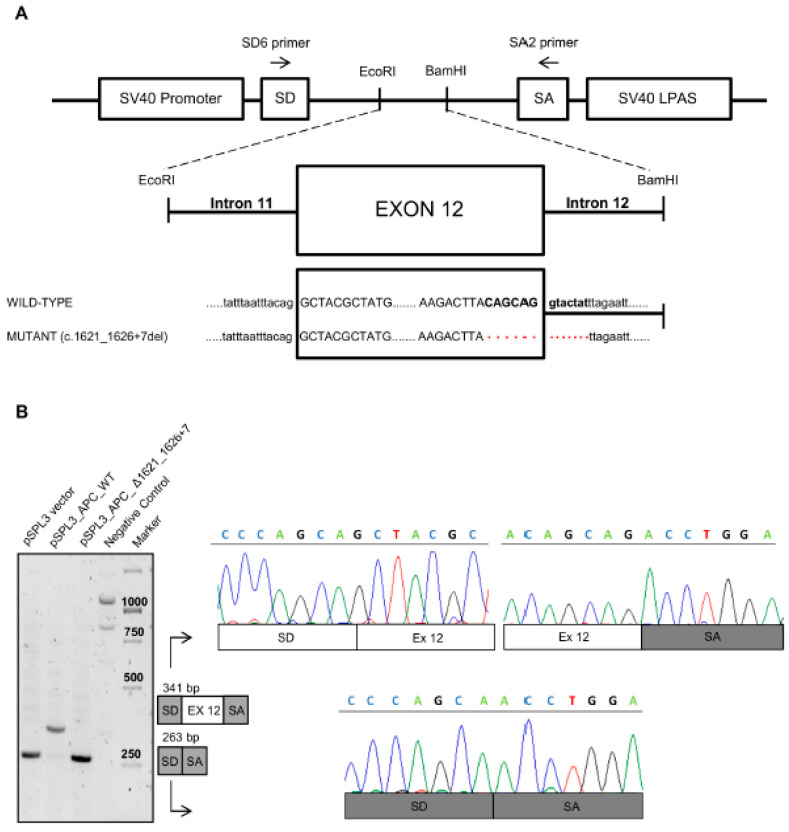
Splicing minigene reporter assay based on the pSPL3 exon-trapping vector. (**A**) Schematic representation of the pSPL3 minigene reporter used for the molecular characterization of *APC* splicing mutation c.1621_1626+7del. The pSPL3 vector contains two exons (SD and SA) and a functional intron, with transcription beginning after the SV40 promoter and ending at the late poly(A) signal (LPAS). EcoRI and BamHI indicate the cloning sites used to subclone the genomic *APC* fragments obtained from the wild type and mutant alleles (c.1621_1626+7del). (**B**) RT-PCR analysis of transcripts derived from the indicated pSPL3 reporter minigenes transfected in HEK-293 cells. Left: Agarose gel electrophoresis showing the RT-PCR products obtained with the SD6 and SA2 primers from HEK-293 cells transfected with the pSPL3 empty vector (263 bp), the pSPL3 vector with the genomic *APC* fragment from the wild type allele (341 bp), or the pSPL3 vector with the genomic *APC* fragment from the mutant allele (263 bp), and untransfected HEK-293 cells (negative control). Center: Schematic diagrams showing the RT-PCR products obtained. Right: Sequencing electropherograms of the RT-PCR products obtained.

To determine the effect of nucleotide deletion c.1621_1626+7del, total RNA was isolated from peripheral blood of the proband and an unrelated control. The *APC* transcript between exons 10 and 14 was amplified by RT-PCR, and the obtained fragments were isolated and sequenced (Figure 2B). Gel electrophoresis of the PCR products showed an expected-size fragment (356 bp) both in control and patient samples; however, a lower fragment (277 bp) was also found in the latter. Sequencing analysis of this fragment showed that the c.1621_1626+7del mutation results in the loss of exon 12 splice donor site and exon 12 skipping (Figure 2B). To confirm our results, we performed a minigene study using the pSPL3 plasmid. As described in the Section 2, fragments with the wild type or mutant exon 12 (78 bp) allele, flanked by upstream (311 nt) and downstream (447 nt) intronic sequences, were cloned into the splicing vector pSPL3 (Figure 3A). The pSPL3 empty vector, pSPL3_APC_wt, and pSPL3_APC_ Δ1621_1626+7 were transfected into HEK-293 cells for 24 hours, and the RNA was collected. Minigene assays showed that the wild type construct resulted in a 341 bp PCR product containing exon 12, while both the empty vector and the mutant construct produced a 263 bp PCR product missing *APC* exon 12. The obtained fragments were confirmed by sequencing analysis (Figure 3B).

### 3.3. Meta-Analysis

We performed a literature meta-analysis of *APC* splicing mutations to identify disease-causing splice site mutations that do not change the reading frame of the aberrant transcript and to evaluate their effect on transcript processing and patient phenotype. So far, 119 unique *APC* splicing mutations, including the one characterized in the present study, have been reported in FAP patients. Of these, 69 (58%) have been molecularly characterized at the mRNA level and mainly (60/69, 87%) cause a reading frame shift, while a very small proportion (9/69, 13%) leads to an in-frame APC protein [39,40,41,42,43,44,45,46,47,48,49,50,51,52,53,54,55,56,57,58,59,60,61,62,63,64,65,66,67,68,69,70,71,72,73,74,75,76,77,78,79,80,81,82,83,84,85,86,87,88] (Table S2). Specifically, among the splicing mutations leading to an in-frame protein, 4/9 cause exon 12 or exon 13 skipping with loss of armadillo repeat 2 (ARM2) and armadillo repeat 3 (ARM3) in the APC N-terminal armadillo repeat domain. Of these, 3 were reported in patients with AFAP phenothype [45,54,59] (Table 1). The remaining molecularly characterized splicing mutations leading to an in-frame protein (5/9) result in skipping of exon 5, 7, 8, or (partially) 9 with loss of APC regions not encompassing known functional sites/domains [45,54,67] (Table S3).

**Figure 4:**
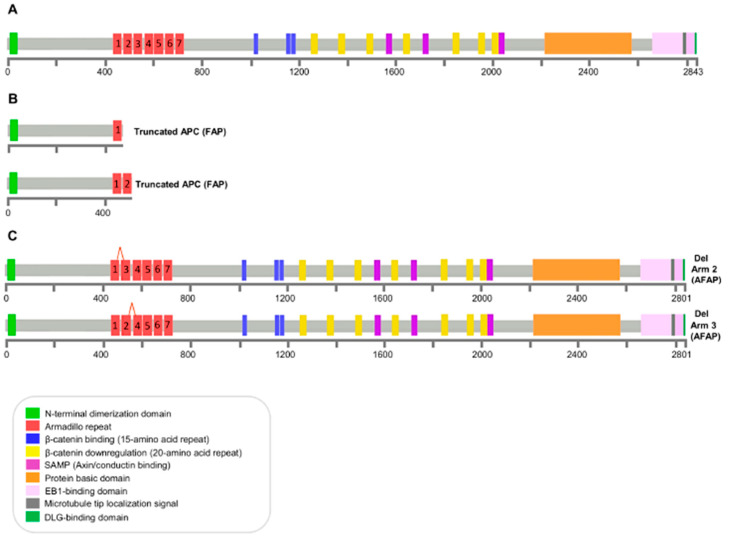
*APC* coding region. (**A**) Schematic diagram of wild type APC protein, depicting conserved regions and domains that interact with other proteins. (**B**) Schematic diagram of APC truncated proteins that result from total removal of ARM2 and/or ARM3 along with disruption of all downstream APC domains and are associated with the classic FAP phenotype. (**C**) Schematic diagram of APC variants that result from exon 12 or exon 13 splicing mutations leading to an in-frame protein and are associated with the AFAP phenotype.

In order to provide further insight into the relationship between *APC* exon 12 or exon 13 splicing mutations leading to an in-frame protein, the clinical phenotype, and the potential underlying molecular mechanisms in FAP disease, we retrieved clinical and molecular data of FAP patients bearing truncating mutations that lead to partial or total removal of ARM2 and/or ARM3 and disrupt all downstream APC protein domains [50,53,71,89,90,91,92,93,94,95,96,97,98,99,100,101,102,103,104,105] (Table S4). Then, we sought to compare the phenotypic consequences of splicing mutations leading to in-frame amino acid deletions within the ARM2 or ARM3 motifs of the APC protein N-terminal armadillo repeat domain with those of truncating mutations located in the ARM2 (aa 505–547) or ARM3 (aa 548–591) domains leading to partial or total removal of ARM2 and/or ARM3 and disrupting all APC downstream regions (aa 505–2843), including the β-catenin-regulating domains (Figure 4). 

A total of 33 patients with data on colon polyposis clinical phenotype and truncating alterations located in the ARM2 and ARM3 domains of APC were identified: (i) 12 patients harbored a truncating mutation involving the ARM2 domain, (ii) four patients harbored a truncating mutation involving the ARM2 and ARM3 domains, and (iii) 17 patients harbored a truncating mutation in the ARM3 domain. In this cohort, the percentage of patients with the classic FAP clinical phenotype was higher (29/33, 87.9%) than the percentage of patients with the attenuated FAP clinical phenotype (4/33, 12.1%). Furthermore, the classic FAP clinical phenotype was only observed in patients with APC truncating mutations, whereas all the patients with splicing mutations leading to in-frame amino acid deletions involving APC ARM2 or ARM3 motifs exhibited the attenuated clinical variant of the disease. 

## 4. Discussion

RNA splicing is a key cellular process that governs several biological processes, including cellular proliferation, survival, and differentiation [106]. Dysregulation of pre-mRNA splicing is increasingly recognized as an important mechanism that is linked to cancer [107]. In the context of multistep carcinogenesis of CRC, genetic lesions that affect *APC* splicing are likely to significantly contribute to the etiology of the disease. 

*APC* is a crucial tumor suppressor gene in both sporadic and hereditary CRC. It encodes a large multifunctional protein comprising several motifs and domains, including an oligomerization domain, an ARM domain, a region containing several β-catenin-binding repeats and axin-binding repeats, and a basic domain that interacts with the microtubules. The wild type APC protein plays an important role in Wnt signaling by promoting the degradation of β-catenin. Due to its interaction with a variety of other proteins, APC is also involved in cellular processes related to cell migration, cell adhesion, proliferation, differentiation, and chromosome segregation [108]. 

*APC* mutational inactivation is a key event in the development of colon cancer and the intestinal polyp disorder FAP. The severity of the FAP phenotype depends on the location of *APC* mutations, indicating a complex role for *APC* that extends beyond the canonical Wnt pathway [9,108]. 

Most *APC* disease-causing variants result in a premature termination codon impairing protein function; however, a minor fraction has been found to disrupt the splicing pattern of the gene [4]. In light of the above, the functional characterization and clinical classification of aberrant splicing variants involving the *APC* gene may support diagnostic accuracy in medical genetics. In this study, we report a novel splicing mutation in the *APC* tumor suppressor gene. This variant was identified by direct sequencing in an Italian AFAP family and consists of a *small* deletion involving the last six nucleotides of exon 12 and seven nucleotides including the splice donor site of intron 12 (c.1621_1626+7del). 

The frequency of this mutation was assessed by interrogating various population databases. This analysis revealed that *APC* c.1621-1626+7del variant is not listed in the dbSNP, 1000 Genome, gnomAD, and ESP databases. To assess the putative effect of this variant on the splicing process, we performed an *in silico* analysis using splicing prediction tools, which indicated a potential splicing alteration due to the loss of *APC* exon 12 canonical splice donor site. 

To confirm whether this mutation could affect *APC* splicing, RNA was isolated from the proband, the *APC* transcript between exon 10 and 14 was amplified by RT-PCR, and the obtained products were isolated and sequenced, revealing the absence of *APC* exon 12. Moreover, to ascertain the *in vivo* relevance of the effect of the identified *APC* mutation on splicing, we performed a minigene splicing assay. Our results showed that *APC* c.1621-1626+7del variant affects the splicing process, resulting in complete skipping of exon 12. However, this deletion does not disrupt the open reading frame of the aberrant transcript, which lacks some, but not all, Armadillo repeat motifs. 

According to ACMG/AMP criteria, our clinical and molecular characterization of the identified variant provides evidence of pathogenicity.

Next, we performed a meta-analysis to investigate the correlation between *APC* exon 12 or exon 13 splicing mutations that lead to an in-frame protein lacking functional domains/sites and the corresponding clinical phenotypes. To date, nearly 2000 *APC* mutations have been described in FAP, almost all of which (~87%) lead to loss of function (nonsense mutations, small deletions, small insertions, and gross rearrangements), while only a few (~6 %) have been reported to cause or potentially cause impaired splicing of the gene product. The remaining *APC* mutations are missense mutations and mutations in regulatory regions of the gene (data obtained from HGMD Professional) [33]. Our literature analysis revealed that only a small proportion of these splice site mutations (69/119) have been characterized at the mRNA level, with the vast majority (60/69, 87%) causing a reading frame shift and a tiny fraction (9/69, 13%) leading to an in-frame APC protein with loss of functional domains/sites (Table S2). 

Specifically, among the molecularly characterized splicing mutations leading to an in-frame protein, 5/9 cause skipping of exon 5, 7, 8, or (partially) 9 with loss of APC regions not encompassing known functional sites/domains, while 4/9 have been reported to cause the deletion of exon 12 (p.Ala517_Gly542del) or 13 (p.Val543_Lys581) with loss of ARM2 and/or ARM3. 

Splicing mutations causing the loss of armadillo functional domains have been reported in patients with FAP disease.

Interestingly, deletion of exon 13 (p.Val543_Lys581del) leads to the loss of the last five amino acids of ARM2 and an almost complete loss of ARM3. 

Skipping of exon 13 has been reported to be associated with a mutation in a highly conserved splice acceptor site (c.1627G>T, the first base of exon 13) in a patient with AFAP phenotype who underwent subtotal colectomy for carcinoma at the age of 60 [54]. In another report, skipping of exon 13 was found to be caused by a missense mutation in exon 13 (c.1742A>G) that was detected in a patient with attenuated FAP [59]. Splicing mutations resulting in the loss of exon 12 (p.Ala517_Gly542del) lead to an almost complete loss of ARM2. Skipping of exon 12 has been reported to be associated with a mutation in a highly conserved splice donor site (c.1626G>C, the last base of exon 12) in a patient with FAP disease whose clinical phenotype was not described [45].

Based on clinical evaluation (age of manifestation, number and size of polyps, and absence of colorectal cancer until the age of 35), the patient carrying the newly identified *APC* splicing mutation c.1621_1627+7del described in this study and its family were classified as having an attenuated form of FAP. 

Patients with AFAP tend to develop fewer adenomatous polyps, with colorectal tumors occurring at an older age compared with patients with classic FAP. Genotype–phenotype association studies have revealed that AFAP patients mainly carry mutations at the 5’ end of the gene or at splice junctions involving the alternatively spliced region of exon 9 [4]. 

Specifically, mutations located in exon 9 alternative splice site have been reported to cause inefficient exon skipping resulting in the generation of a shorter *APC* isoform along with normal transcripts from the mutant allele [39,43,45,47,54,72]. A recent study investigating the molecular mechanisms leading to AFAP in patients carrying a mutation in the alternatively spliced region of exon 9 has suggested that a “third hit” (somatic mutations of both *APC* alleles) is necessary for tumorigenesis to occur in these patients [109]. 

In an effort to elucidate the correlation between mutations causing in-frame loss of functional ARM repeat domains and clinical phenotypes, we compared the clinical and molecular data of FAP patients carrying *APC* splicing mutations that lead to an in-frame protein lacking ARM2 and/or ARM3 with those bearing *APC* truncating mutations that result in partial or total removal of ARM2 and/or ARM3 along with disruption of all downstream domains.

Our results demonstrated a trend towards the development of a milder FAP phenotype (attenuated FAP) in patients with splicing mutations in ARM2 and/or ARM3 compared to patients with truncating mutations. The attenuated phenotype observed in patients harboring *APC* mutations that lead to loss of exon 12 or 13 and cause partial deletion of ARM motifs suggests a potential mechanism of pathogenesis in FAP disease. 

ARM domains are abundant in eukaryotic proteins and are characterized by tandem armadillo repeats of approximately 42 amino acids in length that participate in protein-protein interaction. ARM domains are involved in a broad range of important cellular processes, including signal transduction, nuclear transport, and regulation of cytoskeleton formation [110].

The ARM domain located at APC N-terminal is encoded by exons 10–14 and contains seven armadillo repeats that provide a structural platform for interaction with several other proteins, including SAM68 [111], ASEF [112], KAP3 [113], IQGAP1 [114], and AMER1 [115]. The structural diversity of these binding partners reveals that APC armadillo repeats may be involved in Wnt signaling, cell–cell adhesion, cell polarization, and cell migration. It has also been reported that the loss of ARM domains results in increased tumor initiation, suggesting a putative tumor-suppressive function for this region [116,117]. Furthermore, previous studies on co-crystal structures showed that the replacement of APC key residues, such as N507K, N550K, N594K, and K516/E, abolishes the association between APC-ARM and AMER1-A1/A2/A4 [115], ASEF [118], and SAM68 [111]. These APC ligands show no apparent sequence similarity, nor do they have any resemblance with other APC-binding motifs. However, in their physical interaction with APC, these proteins occupy the same surface groove within APC-ARM domains and assume the same antiparallel position with respect to armadillo repeats [115]. 

The evidence that addition of the ASEF protein to preassembled APC-ARM/A MER1 complexes progressively dissociates APC-ARM from AMER1 in a dose-dependent manner confirms the hypothesis that these APC partners can compete with each other in a mutually exclusive manner [115].

Consistently, from a functional point of view, AMER1 and SAM68 seem to have antithetical roles in the regulation of the Wnt pathway. In particular, AMER1 negatively regulates Wnt signal transduction by promoting ubiquitination and degradation of β-catenin [119], while recent data showed that aberrant upregulation of SAM68 induces cancer cell proliferation in vitro by activating the Wnt/β-catenin signaling pathway [120]. Note that it has also been reported that the complex between APC and SAM68 regulates the alternative splicing of members of the T cell factor (TCF) family of transcription factors that associate with β-catenin, in the presence of the Wnt signal or in the absence of APC, in order to regulate the expression of Wnt target genes involved in tumor formation. *APC* mutations that truncate regions downstream of the ARM domain lead to the accumulation of the TCF-1E splice variant, which strongly transactivates Wnt target genes [111]. 

Note also that genetic disease mutations can have an impact on protein conformational equilibria and dynamics [121,122]. Recently, the missense *APC* N1026S variant identified in an AFAP family has been predicted to change the conformational flexibility of APC protein, preventing it from establishing stable contacts with β-catenin protein [123]. 

In this light, another possible explanation as to why mutations causing partial deletion of APC ARM1 and/or ARM2 motifs could produce an attenuated phenotype is that these deletions may induce conformational changes in protein structure and dynamics resulting in impaired physical interaction between APC and its binding partners, including β-catenin. It is therefore tempting to speculate that APC exon 12 or exon 13 splicing mutations leading to the deletion of ARM motifs may decrease APC binding to β-catenin, thereby preventing, at least in part, the export of nuclear β-catenin. This would in turn result in an attenuated clinical variant of FAP disease.Conversely, truncating mutations located in the ARM2 and/or ARM3 motifs lead to complete lack of regulation of the β-catenin protein, causing the classic clinical variant of FAP disease. 

For these reasons, *APC* exon 12 or exon 13 splicing mutations leading to the partial loss of ARM motifs are expected to account for the observed AFAP phenotype in the patients considered in the present study. 

## 5. Conclusions

Altogether, the presented evidence supports mechanism for FAP pathogenesis involving mutations that affect APC ARM domains but do not cause the loss of the seven β-catenin-downregulating 20 amino acid repeats distributed in the central region of the protein. The underlying mechanism of pathogenesis most likely involves binding partners and functions of APC ARM motifs, suggesting that these domains mediate APC tumor suppressor activity and may play a role in carcinogenesis in FAP patients.In conclusion, our findings support a pathogenic role for *APC* exon 12 or exon 13 splicing mutations in the AFAP phenotype. 

## Figures and Tables

**Table 1:** *APC* splicing mutations leading to an in-frame protein and skipping of exon 12 or exon 13.

Gene	Variant (Human Genome Variation Society, HGVS)	Chromosome Position (GRCh37)	Location	Observed Effect on splicing	Effect on mRNA (HGVS)	Effect on Protein (HGVS)	Clinical Phenotype (Classic FAP/AFAP)	Reference
*APC*	c.1621_1626+7del	chr5:g.112163698- 112163710del	Exon 12/ Intron 12	Exon 12 skipping	r.1549_1626del	p.A517_Q542del	AFAP	Present study
*APC*	c.1626G>C	chr5:g.112164552G>C	Exon 12	Exon 12 skipping	r.1549_1626del	p.A517_Q542del	n.d.	[45]
*APC*	c.1627G>T	chr5:g.112164553G>T	Exon 13	Exon 13 skipping	r.1627_1743del	p.V543_K581del	AFAP	[54]
*APC*	c.1742A>G	chr5:g.112164668A>G	Exon 13	Exon 13 skipping	r.1627_1743del	p.V543_K581del	AFAP	[59]

n.d.: not discriminated.

## Supplementary Materials

The following supplementary figure and tables are available online at https://www.mdpi.com/2073-4425/12/3/353/s1. Figure S1: Screenshot from the Alamut software. Splicing effect window around *APC* c.1621_1626+7del mutation. The top box represents *APC* wild-type sequence; the bottom box represents *APC* mutated sequence with the c.1621_1626+7del deletion. The dark blue bars represent the predicted splice donor site. The diagram reveals the abolition of the canonical splice donor site at position c.1626. All four tools predicted that the identified variant abolishes the canonical splice donor site. Table S1: Primers used for genomic PCR amplification and sequencing reactions; Table S2: *APC* splicing mutations; Table S3: *APC* splicing mutations leading to an in-frame protein and loss of APC regions not encompassing known functional sites/domains; Table S4: Truncating mutations located in the ARM2 or ARM3 domain of the APC protein.
