# Supplementary material for: APC Splicing Mutations Leading to In-Frame Exon 12 or Exon 13 Skipping Are Rare Events in FAP Pathogenesis and Define the Clinical Outcome
Source: Genes (Basel). 2021 Feb 28;12(3):353. doi: 10.3390/genes12030353 (PMC7997234; doi:10.3390/genes12030353)
Supplement: Supplementary file 1 [file genes-12-00353-s001.zip › genes-1080592/Supplementary Table_2.docx]

Supplementary Table 2. *APC* splicing mutations

| Gene | Variant  (Human Genome Variation Society, HGVS) | Chromosome position (GRCh37) | Location | Observed effect on splicing using functional assays  (effect on mRNA, HGVS) | Observed effect on protein (HGVS) | Reference |
| --- | --- | --- | --- | --- | --- | --- |
| *APC* | c.220+2T>A | chr5:g.112102109T>A | Intron 2 | n.e. | p.? | [39] |
| *APC* | c.221-2A>G | chr5:g.112102884A>G | Intron 2 | n.e. | p.? | [39,40] |
| *APC* | c.221-1G>A | chr5:g.112102885G>A | Intron 2 | n.a. | Premature stop codon (p.Glu74GlyfsX29) | [41] |
| *APC* | c.221-14T>G | chr5:g.102102872T>G | Intron 3 | n.e. | p.? | [42] |
| *APC* | c.422+1G>A | chr5:g.112103088G>A | Intron 3 | n.e. | p.? | [39] |
| *APC* | c.422+2T>A | chr5:g.112103089T>A | Intron 3 | n.e. | p.? | [43] |
| *APC* | c.422+2T>G | chr5:g.112103089T>G | Intron 3 | n.e. | p.? | [39] |
| *APC* | c.423-12A>G | chr5:g.112111314A>G | Intron 3 | Splice acceptor site moved up by 11 nucleotides | Premature stop codon (p.Ser142LysfsX32) | [44] |
| *APC* | c.423-11A>G | chr5:g.112111315A>G | Intron 3 | Insertion of the last 10 nucleotides of intron 3 | Premature stop codon | [45] |
| *APC* | c.423-9A>G | chr5:g.112111317A>G | Intron 3 | Insertion of the last 8 nucleotides of intron 3 | Premature stop codon | [45] |
| *APC* | c.423-6A>G | chr5:g.112111320A>G | Intron 3 | Shift of the first nucleotide of the exon by  five positions towards the 5' region | Premature stop codon | [46] |
| *APC* | c.423-5A>G | chr5:g.112111321A>G | Intron 3 | Exon 4 skipping (r.423_531del) | Premature stop codon (p.Arg144SerfsX8) | [47] |
| *APC* | c.423-3T>A | chr5:g.112111323T>A | Intron 3 | n.e. | p.? | [39,48] |
| *APC* | c.423-2A>C | chr5:g.112111324A>C | Intron 3 | n.e. | p.? | [49] |
| *APC* | c.423-2A>G | chr5:g.112111324A>G | Intron 3 | n.e. | p.? | [50] |
| *APC* | c.423-2A>T | chr5:g.112111324A>T | Intron 3 | n.e. | p.? | [47] |
| *APC* | c.423-1G>A | chr5:g.112111325G>A | Intron 3 | Exon 4 skipping (r.423_531del) | Premature stop codon (p.Arg144SerfsX8) | [51] |
| *APC* | c.423-1G>C | chr5:g.112111325G>C | Intron 3 | Exon 4 skipping (r.423_531del) | Premature stop codon (p.Arg144SerfsX8) | [47] |
| *APC* | c.423-1G>T | chr5:g.112111325G>T | Intron 3 | Exon 4 skipping (r.423_531del) | Premature stop codon (p.Arg144SerfsX8) | [47] |
| *APC* | c.423-1delG | chr5:g.112111325delG | Intron 4 | Exon 4 skipping (r.423_531del) | Premature stop codon (p.Arg141SerfsX8) | [44] |
| *APC* | c.423G>T | chr5:g.112111326G>T | Exon 4 | Exon 4 skipping (r.423_531del) | Premature stop codon (p.Arg144SerfsX8) | [45,47] |
| *APC* | c.531+1G>A | chr5:g.112111435G>A | Intron 4 | Exon 4 skipping (r.423_531del) | Premature stop codon (p.Arg144SerfsX8) | [47,52] |
| *APC* | c.531+1G>T | chr5:g.112111435G>T | Intron 4 | Exon 4 skipping (r.423_531del) | Premature stop codon (p.Arg144SerfsX8) | [53,54] |
| *APC* | c.531+2T>A | chr5:g.112111436T>A | Intron 4 | n.e. | p.? | [55] |
| *APC* | c.531+2T>C | chr5:g.112111436T>C | Intron 4 | n.e. | p.? | [50] |
| *APC* | c.531+3A>C | chr5:g.112111437A>C | Intron 4 | n.e. | p.? | [39,56] |
| *APC* | c.531+5G>A | chr5:g.112111439G>A | Intron 4 | n.e. | p.? | [57,58] |
| *APC* | c.531+5G>C | chr5:g.112111439G>C | Intron 4 | Exon 4 skipping (r.423_531del) | Premature stop codon (p.Arg144SerfsX8) | [52,59] |
| *APC* | c.532-941G>A | chr5:g.112115548G>A | Intron 4 | Insertion of 167 nucleotides | Premature stop codon (p.Phe178ArgfsX22) | [60,61] |
| *APC* | c.532-8G>A | chr5:g.112116479G>A | Intron 4 | Insertion of the last 6 nucleotides of intron 4 | Premature stop codon | [45,59] |
| *APC* | c.532-2A>C | chr5:g.112116485A>C | Intron 4 | n.e. | p.? | [62] |
| *APC* | c.532-2A>G | chr5:g.112116485A>G | Intron 4 | n.e. | p.? | [48] |
| *APC* | c.532-2A>T | chr5:g.112116485A>T | Intron 4 | n.e. | p.? | [63] |
| *APC* | c.532-1G>A | chr5:g.112116486G>A | Intron 4 | n.a. | Premature stop codon | [64] |
| *APC* | c.532-1G>T | chr5:g.112116486G>T | Intron 4 | n.a. | Premature stop codon | [64] |
| *APC* | c.645+1G>A | chr5:g.112116601G>A | Intron 5 | n.e. | p.? | [43] |
| *APC* | c.645+1G>T | chr5:g.112116601G>T | Intron 5 | Exon 5 skipping (r.532_645del) | In-frame protein  (p.Phe178_Gln215del) | [53,54] |
| *APC* | c.645+2T>G | chr5:g.112116602T>G | Intron 5 | n.e. | p.? | [65] |
| *APC* | c.646-8T>A | chr5:g.112128135T>A | Intron 5 | n.e. | p.? | [57] |
| *APC* | c.646-2A>C | chr5:g.112128141A>C | Intron 5 | n.e. | p.? | [58] |
| *APC* | c.646-2A>G | chr5:g.112128141A>G | Intron 5 | Deletion of 5 nucleotides of exon 6 (r.646_650del) | Premature stop codon | [66,67] |
| *APC* | c.646-1G>A | chr5:g.112128142G>A | Intron 5 | n.e. | p.? | [65] |
| *APC* | c.646-1G>T | chr5:g.112128142G>T | Intron 5 | n.e. | p.? | [55] |
| *APC* | c.730-3C>G | chr5:g.112136973C>G | Intron 6 | Exon 7 skipping (r.730_834del) | In-frame protein  (p.Arg244_Gln278del) | [45,55] |
| *APC* | c.730-1G>T | chr5:g.112136975G>T | Intron 6 | Loss of the normal splice site sequence and use  of a cryptic splice site 2 nucleotides downstream | Premature stop codon | [43,47] |
| *APC* | c.832C>A | chr5:g.112137078C>A | Exon 7 | This substitution results in the creation of a new splice donor site 5 nucleotides upstream | Premature stop codon (p.Gln278PhefsX8) | [44] |
| *APC* | c.834G>A | chr5:g.112137080G>A | Intron 7 | n.e. | p.? | [39,48] |
| *APC* | c.834G>C | chr5:g.112137080G>C | Exon 7 | This mutation leads to the use of a cryptic splice donor site 11 nucleotides upstream in exon 7 | Premature stop codon | [68] |
| *APC* | c.834+2T>C | chr5:g.112137082T>C | Intron 7 | This mutation induces aberrant transcription and produces a truncated APC protein | Premature stop codon | [69] |
| *APC* | c.834+1G>A | chr5:g.112137081G>A | Intron 7 | 11-nucleotide frameshift deletion (r.825_835del) | Premature stop codon (p.Gly275GlyfsX9) | [43,63,66,70] |
| *APC* | c.835-17A>G | chr5:g.112151175A>G | Intron 7 | Insertion of the last 16 nucleotides of intron 7 between exon 7 and 8 sequences, adding two novel amino acids and a stop codon at position 281 | Premature stop codon | [43,63,70,71] |
| *APC* | c.835-8A>G | chr5:g.112151184A>G | Intron 7 | Insertion of 7 nucleotides due to the creation of a new AG splice acceptor site 7 nucleotides upstream of the actual site | Premature stop codon | [72] |
| *APC* | c.835-7T>G | chr5:g.112151185T>G | Intron 7 | Introduction of a new active splice site 6 nucleotides upstream of the wild-type AG splice site of intron 7 | Premature stop codon | [68] |
| *APC* | c.835-3T>C | chr5:g.112151189T>C | Intron 7 | n.e. | p.? | [57] |
| *APC* | c.835-1G>A | chr5:g.112151190G>A | Intron 7 | This substitution results in the use of a cryptic splice acceptor site immediately adjacent to the mutation, which creates an apparent “G” deletion at the transcriptional level | Premature stop codon  (p.Ser293Stop) | [43,73] |
| *APC* | c.933G>C | chr5:g.112151290G>C | Exon 8 | Exon 8 skipping (r.835_933del) | In-frame protein  (p.Gly279_Lys311del) | [67] |
| *APC* | c.933+1G>A | chr5:g.112151291G>A | Intron 8 | n.e. | p.? | [67] |
| *APC* | c.933+2T>C | chr5:g.112151292T>C | Intron 8 | n.e. | p.? | [47,67] |
| *APC* | c.1230G>A | chr5:g.112154959G>A | Intron 9 | n.e. | p.? | [45] |
| *APC* | c.1234C>T | chr5:g.112154963C>T | Exon 9 | Exon 9 skipping | Premature stop codon (p.Gln412X) | [72] |
| *APC* | c.1240C>T | chr5:g.112154969C>T | Exon 9 | Exon 9 partial skipping (r.934_1236del) | In-frame protein deletion (p.Val312_Gln412del) | [67] |
| *APC* | c.1242C>T | chr5:g.112154971C>T | Exon 9 | Exon 9 partial skipping (r.934_1236del) | In-frame protein deletion (p.Val312_Gln412del) | [67] |
| *APC* | c.1312+1G>A | chr5:g.112155042G>A | Intron 9 | n.e. | p.? | [74] |
| *APC* | c.1312+1G>C | chr5:g.112155042G>C | Intron 9 | n.e. | p.? | [39] |
| *APC* | c.1312+2T>C | chr5:g.112155043T>C | Intron 9 | n.e. | p.? | [48] |
| *APC* | c.1312+2T>G | chr5:g.112155043T>G | Intron 9 | n.e. | p.? | [75] |
| *APC* | c.1312+3A>C | chr5:g.112155044A>C | Intron 9 | n.e. | p.? | [76] |
| *APC* | c.1312+3A>G | chr5:g.112155044A>G | Intron 9 | Exon 9 skipping (r.934_1312del) / Exon 9 partial skipping (r.934_1236del) | Premature stop codon (p.Gln412X) / In-frame protein deletion (p.Val312_Gln412del) | [39,43,45,47] |
| *APC* | c.1312+5G>A | chr5:g.112155046G>A | Intron 9 | Exon 9 skipping (r.934_1312del) / Exon 9 partial skipping (r.934_1236del) | Premature stop codon (p.Gln412X) / In-frame protein deletion (p.Val312_Gln412del) | [47,54] |
| *APC* | c.1312+5G>C | chr5:g.112155046G>C | Intron 9 | n.e. | p.? | [39] |
| *APC* | c.1312+5G>T | chr5:g.112155046G>T | Intron 9 | Exon 9 skipping (r.934_1312del) | Premature stop codon (p.Gln412X) | [21,45] |
| *APC* | c.1313-1G>C | chr5:g.112157592G>C | Intron 9 | n.e. | p.? | [42] |
| *APC* | c.1409-6A>G | chr5:g.112162799A>G | Intron 10 | Insertion of the last 5 nt of intron 10 | Premature stop codon | [45] |
| *APC* | c.1409-5A>G | chr5:g.112162800A>G | Intron 10 | Exon 11 skipping (r.1409_1548del) | Premature stop codon (p.Gly470GlyfsX20) | [45] |
| *APC* | c.1409-3T>G | chr5:g.112162802T>G | Intron 10 | n.e. | p.? | [67] |
| *APC* | c.1409-2A>C | chr5:g.112162803A>C | Intron 10 | n.e. | p.? | [47] |
| *APC* | c.1409-2A>G | chr5:g.112162803A>G | Intron 10 | Exon 11 skipping (r.1409_1548del) | Premature stop codon (p.Gly470GlyfsX20) | [47] |
| *APC* | c.1409-1G>A | chr5:g.112162804G>A | Intron 10 | New splice acceptor site 1 nucleotide downstream | Premature stop codon | [47,64] |
| *APC* | c.1409-1G>T | chr5:g.112162804G>T | Intron 10 | n.e. | p.? | [67] |
| *APC* | c.1458T>C | chr5:g.112162854T>C | Exon 11 | Exon 11 skipping (r.1409_1548del) | Premature stop codon (p.Gly470GlyfsX20) | [77] |
| *APC* | c.1408+731C>T | chr5:g.112158419C>T | Intron 10 | New splice donor site | Premature stop codon (p.Gly471SerfsX55) | [60] |
| *APC* | c.1408+735A>T | chr5:g.112158423A>T | Intron 10 | n.e. | p.? | [60] |
| *APC* | c.1549-8A>C | chr5:g.112163618A>C | Intron 11 | Insertion of the last 7 nucleotides of intron 11 | Premature stop codon | [45,78] |
| *APC* | c.1549-1G>A | chr5:g.112163625G>A | Intron 11 | n.e. | p.? | [48] |
| *APC* | c.1548G>A | chr5:g.112162944G>A | Exon 11 | Exon 11 skipping (r.1409_1548del) | Premature stop codon (p.Gly470GlyfsX20) | [59] |
| *APC* | c.1548G>C | chr5:g.112162944G>C | Exon 11 | n.e. | p.? | [65] |
| *APC* | c.1548G>T | chr5:g.112162944G>T | Exon 11 | Exon 11 skipping (r.1409_1548del) | Premature stop codon (p.Gly470GlyfsX20) | [67] |
| *APC* | c.1548+1G>A | chr5:g.112162945G>A | Intron 11 | Exon 11 skipping (r.1409_1548del) | Premature stop codon (p.Gly470GlyfsX20) | [54,79] |
| *APC* | c.1548+1G>T | chr5:g.112162945G>T | Intron 11 | Exon 11 skipping (r.1409_1548del) | Premature stop codon (p.Gly470GlyfsX20) | [54,79] |
| *APC* | c.1621_1626+7del | Chr5:g.112163698-112163710del | Exon 12/  Intron 12 | Exon 12 skipping (r.1549_1626del) | In-frame protein deletion (p.Ala517_Gln542del) | Present Study |
| *APC* | c.1626G>C | chr5:g.112164552G>C | Exon 12 | Exon 12 skipping (r.1549_1626del) | In-frame protein deletion (p.Ala517_Gln542del) | [45] |
| *APC* | c.1627G>T | chr5:g.112164553G>T | Exon 13 | Exon 13 skipping (r.1627_1743del) | In-frame protein deletion (p.Val543_Lys581del) | [54] |
| *APC* | c.1744-3T>G | chr5:g.112170645T>G | Intron 13 | n.e. | p.? | [42] |
| *APC* | c.1744-2A>G | chr5:g.112170646A>G | Intron 13 | Exon 14 skipping (r.1744_1958del) | Premature stop codon | [47,65] |
| *APC* | c.1744-1G>A | chr5:g.112170647G>A | Intron 13 | n.e. | p.? | [80] |
| *APC* | c.1744G>T | chr5:g.112170648G>T | Exon 14 | Exon 14 skipping (r.1744_1958del) | Premature stop codon | [81] |
| *APC* | c.1742A>G | chr5:g.112164668A>G | Intron 13 | Exon 13 skipping (r.1627_1743del) | In-frame protein deletion (p.Val543_Lys581del ) | [59] |
| *APC* | c.1743+1G>A | chr5:g.112164670G>A | Intron 13 | n.e. | p.? | [42] |
| *APC* | c.1743+1G>T | chr5:g.112164670G>T | Intron 13 | n.e. | p.? | [67] |
| *APC* | c.1743+2T>C | chr5:g.112164671T>C | Intron 13 | n.e. | p.? | [65] |
| *APC* | c.1869G>T | chr5:g.112170775G>T | Exon 14 | Exon 14 skipping (r.1744_1958del) | Premature stop codon (p.Gly470GlyfsX20) | [82] |
| *APC* | c.1958+1G>A | chr5:g.112170863G>A | Intron 14 | Exon 14 skipping (r.1744_1958del) | Premature stop codon (p.Gly470GlyfsX20) | [66] |
| *APC* | c.1959-6T>G | chr5:g.112173244T>G | Intron 14 | Insertion of the last 5 nucleotides of intron 14 | Premature stop codon | [45] |
| *APC* | c.1959-1G>A | chr5:g.112173249G>A | Intron 14 | n.e. | p.? | [67] |
| *APC* | c.1902T>G | chr5:g.112170806T>G | Exon 14 | Exon 14 skipping (r.1744_1958del) | Premature stop codon (p.Gly470GlyfsX20) | [40,83] |
| *APC* | c.1918C>G | chr5:g.112170822C>G | Exon 14 | Exon 14 skipping (r.1744_1958del) | Premature stop codon (p.Gly470GlyfsX20) | [84,85] |
| *APC* | c.1956C>T | chr5:g.112170860C>T | Exon 14 | Exon 14 skipping (r.1744_1958del) | Premature stop codon (p.Gly470GlyfsX20) | [47,54] |
| *APC* | c.1957A>C | chr5:g.112170861A>C | Exon 14 | Exon 14 skipping (r.1744_1958del) | Premature stop codon (p.Gly470GlyfsX20) | [47] |
| *APC* | c.1957A>G | chr5:g.112170861A>G | Exon 14 | Exon 14 skipping (r.1744_1958del) | Premature stop codon (p.Gly470GlyfsX20) | [45,47] |
| *APC* | c.1957A>T | chr5:g.112170861A>T | Exon 14 | Exon 14 skipping (r.1744_1958del) | Premature stop codon (p.Gly470GlyfsX20) | [67] |
| *APC* | c.1958G>A | chr5:g.112170862G>A | Exon 14 | Exon 14 skipping (r.1744_1958del) | Premature stop codon (p.Gly470GlyfsX20) | [45,67] |
| *APC* | c.1958G>C | chr5:g.112170862G>C | Exon 14 | Exon 14 skipping (r.1744_1958del) | Premature stop codon (p.Gly470GlyfsX20) | [86,87] |
| *APC* | c.1958G>T | chr5:g.112170862G>T | Exon 14 | n.e. | p.? | [39,88] |
| *APC* | c.1958+1G>A | chr5:g.112170863G>A | Intron 14 | Exon 14 skipping (r.1744_1958del) | Premature stop codon (p.Gly470GlyfsX20) | [47] |
| *APC* | c.1958+1G>T | chr5:g.112170863G>T | Intron 14 | Exon 14 skipping (r.1744_1958del) | Premature stop codon (p.Gly470GlyfsX20) | [47] |
| *APC* | c.1958+2T>C | chr5:g.112170864T>C | Intron 14 | n.e. | p.? | [67] |
| *APC* | c.1958+2T>G | chr5:g.112170864T>G | Intron 14 | n.e. | p.? | [64] |
| *APC* | c.1958+3A>C | chr5:g.112170865A>C | Intron 14 | Exon 14 skipping (r.1744_1958del) | Premature stop codon (p.Gly470GlyfsX20) | [67] |
| *APC* | c.1958+3A>G | chr5:g.112170865A>G | Intron 14 | Exon 14 skipping (r.1744_1958del) | Premature stop codon (p.Gly470GlyfsX20) | [45,47] |

n.e.: effect on splicing not evaluated by functional assays; p.?: effect on protein unknown; n.a.: effect on mRNA splicing not available
