# Supplementary material for: APC Splicing Mutations Leading to In-Frame Exon 12 or Exon 13 Skipping Are Rare Events in FAP Pathogenesis and Define the Clinical Outcome
Source: Genes (Basel). 2021 Feb 28;12(3):353. doi: 10.3390/genes12030353 (PMC7997234; doi:10.3390/genes12030353)
Supplement: Supplementary file 1 [file genes-12-00353-s001.zip › genes-1080592/Supplementary Table_4.docx]

Supplementary Table 4. Truncating mutations located in the ARM2 or ARM3 domain of the APC protein.

| Gene | Variant (Human Genome Variation Society, HGVS) | Location | APC protein change | Protein domain | Family subjects | Colon polyposis phenotype  (age at Dx) | Polyp number | CRC (age at Dx) | Reference |
| --- | --- | --- | --- | --- | --- | --- | --- | --- | --- |
| *APC* | c.1557_1561delATGCT | Exon 12 | p.(Cys520Tyrfs*15) | ARM 2 | F1-I | AFAP | n.a. | n.a. | [71] |
|  |  |  |  |  | F1-II | AFAP | n.a. | n.a. |  |
|  |  |  |  |  | F1-III | AFAP | n.a. | n.a. |  |
| *APC* | c.1576dupA | Exon 12 | p.(Met526Asnfs*11) | ARM 2 | F2-I | Classic FAP | >100 | n.a. | [89] |
| *APC* | c.1586_1587insAT | Exon 12 | p.(Val530Leufs*5) | ARM 2 | F3-I | Classic FAP | >100 | Yes  (44) | [90] |
| *APC* | c.1594delC | Exon 12 | p.(Gln532Asnfs*2) | ARM 2 | F4-I | Classic FAP | >100 | n.a. | [91] |
| *APC* | c.1594delC | Exon 12 | p.(Gln532Asnfs*2) | ARM 2 | F5-I | Classic FAP  (20-29) | >100 | n.a. | [92] |
| *APC* | c.1602dupA | Exon 12 | p.(Ser535Ilefs*2) | ARM 2 | F6-I | Classic FAP | >100 | n.a. | [89] |
| *APC* | c.1605_1606delTG | Exon 12 | p.(Glu536Lysfs*2) | ARM 2 | F7-I | Classic FAP (54) | >100 | n.a. | [93] |
|  |  |  |  |  | F7-II | Classic FAP (28) | >100 | n.a. |  |
|  |  |  |  |  | F7-III | Classic FAP (18) | >100 | n.a. |  |
| *APC* | c.1609delA | Exon 12 | p.(Ser537Valfs*12) | ARM 2/  ARM3 | F8-I | Classic FAP | >100 | n.a. | [94] |
| *APC* | c.1614dupA | Exon 12 | p.(Asp539Argfs*21) | ARM 2/  ARM3 | F9-I | Classic FAP | >100 | n.a. | [95] |
| *APC* | c.1620dupA | Exon 12 | p.(Gln541Thrfs*19) | ARM 2/  ARM3 | F10-I | Severe FAP (10) | n.a. | n.a. | [96] |
| *APC* | c.1622dupA | Exon 12 | p.(Gln542Alafs*18) | ARM 2/  ARM3 | F11-I | Classic FAP | > 100 | n.a. | [50] |
| *APC* | c.1624C>T | Exon 12 | p.Q542* | ARM 2 | F12-I | Classic FAP | > 100 | n.a. | [53] |
| *APC* | c.1629delT | Exon 13 | p.(Ile544Leufs*5) | ARM 3 | F13-I | Classic FAP (42) | >100 | Yes  (42) | [97] |
| *APC* | c.1636_1639delAGTG | Exon 13 | p.(Ser546Phefs*2) | ARM 3 | F14-I | Classic FAP (41) | >100 | Yes  (41) | [98] |
| *APC* | c.1642_1643delTT | Exon 13 | p.(Leu548Glufs*11) | ARM 3 | F15-I | Classic FAP (27) | 100–1000 | No | [99] |
| *APC* | c.1659G>A | Exon 13 | p.W553* | ARM 3 | F16-I | Classic FAP (39) | >100 | No | [100] |
|  |  |  |  |  | F16-II | Classic FAP (46) | >500 | No |  |
| *APC* | c.1660C>T | Exon 13 | p.R554* | ARM 3 | F17-I | Profuse FAP (19) | >1000 | n.a. | [101] |
| *APC* | c.1673delA | Exon 13 | p.(Asn558Ilefs*12) | ARM 3 | F18-I | Classic FAP (43) | >100 | Yes  (43) | [102] |
| *APC* | c.1699G>T | Exon 13 | p.G567* | ARM 3 | F19-I | Classic FAP (25) | >100 | No | [103] |
|  |  |  |  |  | F20-I | AFAP | <100 | n.a. | [71] |
| *APC* | c.1732G>T | Exon 13 | p.E578* | ARM 3 | F21-I | Classic FAP | >100 | n.a. | [104] |
| *APC* | c.1682dupA | Exon 13 | p.(Thr562Aspfs*19) | ARM 3 | F22-I | Severe FAP (7) | n.a. | n.a. | [105] |
|  |  |  |  |  | F22-II | Severe FAP | n.a. | n.a. |  |
|  |  |  |  |  | F22-III | Severe FAP | n.a. | n.a. |  |
|  |  |  |  |  | F22-IV | Severe FAP | n.a. | n.a. |  |
|  |  |  |  |  | F22-V | Severe FAP | n.a. | n.a. |  |
|  |  |  |  |  | F22-VI | Severe FAP | n.a. | n.a. |  |
|  |  |  |  |  | F22-VII | Severe FAP | n.a. | n.a. |  |

CRC: colorectal cancer; Dx: diagnosis; n.a.: not available; Yes: presence of clinical phenotype; No: absence of clinical phenotype
