# Supplementary material for: APC Splicing Mutations Leading to In-Frame Exon 12 or Exon 13 Skipping Are Rare Events in FAP Pathogenesis and Define the Clinical Outcome
Source: Genes (Basel). 2021 Feb 28;12(3):353. doi: 10.3390/genes12030353 (PMC7997234; doi:10.3390/genes12030353)
Supplement: Supplementary file 1 [file genes-12-00353-s001.zip › genes-1080592/Supplementary_Figure_1.pdf]

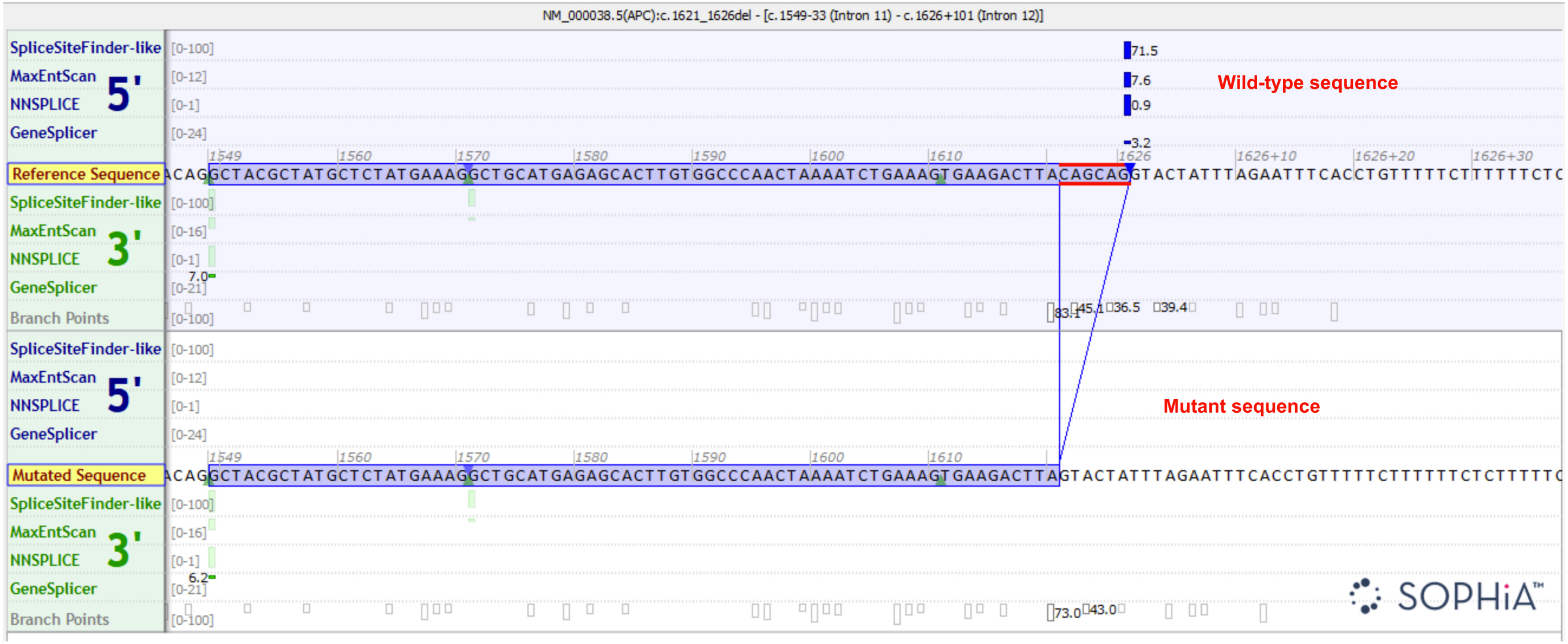

Donor Sites

	SSF [0-100]	MaxEnt [0-12]	NNSPLICE [0-1]	GeneSplicer [0-24]
Threshold	*≥ 70*	*≥ 0*	*≥ 0.4*	*≥ 0*
**Exon 12 – c.1626 N**	**71.49 ⇒ —**	**7.64 ⇒ —**	**0.90 ⇒ —**	**3.17 ⇒ —**
