# Supplementary material for: APC Splicing Mutations Leading to In-Frame Exon 12 or Exon 13 Skipping Are Rare Events in FAP Pathogenesis and Define the Clinical Outcome
Source: Genes (Basel). 2021 Feb 28;12(3):353. doi: 10.3390/genes12030353 (PMC7997234; doi:10.3390/genes12030353)
Supplement: Supplementary file 1 [file genes-12-00353-s001.zip › genes-1080592/Supplementary_Table_1.docx]

Supplementary Table 1. Primers used for genomic PCR amplification and sequencing reactions

| **Exon** | **Forward Primer** | **Reverse Primer** | **PCR/Sequencing** |
| --- | --- | --- | --- |
| 1 | AGGTCCAAGGGTAGCCAAGG | TAAAAATGGATAAACTACAATAAAAG | PCR and Sequencing |
| 2 | AAATACAGAATCATGTCTTGAAGT | ACACCTAAAGATGACAATTTGAG | PCR and Sequencing |
| 3 | TAACTTAGATAGCAGTAATTTCCC | ACAATAAACTGGAGTACACAAGG | PCR and Sequencing |
| 4 | ATAGGTCATTGCTTCTTGCTGAT | TGAATTTTAATGGATTACCTAGGT | PCR and Sequencing |
| 5 | CTTTTTTTGCTTTTACTGATTAACG | TGTAATTCATTTTATTCCTAATAGCTC | PCR and Sequencing |
| 6 | GGTAGCCATAGTATGATTATTTCT | CTACCTATTTTTATACCCACAAAC | PCR and Sequencing |
| 7 | AAGAAAGCCTACACCATTTTTTG | GATCATTCTTAGAACCATCTTGC | PCR and Sequencing |
| 8 | ACCTATAGTCTAAATTATACCATC | GTCATGGCATTAGTGACCAG | PCR and Sequencing |
| 9 | AGTCGTAATTTTGTTTTCTAAACTC | TGAAGGACTGGATTTCACGC | PCR and Sequencing |
| 9A | TCATTCACTCACAGCCTGATGAC | GCTTTGAAACATGCACTACGAT | PCR and Sequencing |
| 10 | AAACATCATTGCTCTTCAAATAAC | TACCTGATTTAAAATCCACCAG | PCR and Sequencing |
| 11 | GATGATTGTCTTTTCCTCTTGC | CTGAGCTATCTTAAGAAATACATG | PCR and Sequencing |
| 12 | TTTTAAATGATCCTCTATTCTGTAT | ACAGAGTCAGACCCTGCCTCAAAG | PCR and Sequencing |
| 13 | TTTCTATTCTTACTGCTAGCATT | ATACACAGGTAAGAAATTAGGA | PCR and Sequencing |
| 14 | TAGATGACCCATATTCTGTTTC | CAATTAGGTCTTTTTGAGAGTA | PCR and Sequencing |
| 15A | GTTACTGCATACACATTGTGAC | TGTTTGGGTCTTGCCCATCTT | PCR and Sequencing |
| 15B | AGTCCCATACACATTCAAACAC | ATGAGTGGGGTCTCCTGAAC | PCR and Sequencing |
| 15C | ATCTCCCTCCAAAAGTGGTGC | GAGCCTCATCTGTACTTCTGC | PCR and Sequencing |
| 15D | CCCTCCAAATGAGTTAGCTGC | ATTGTGTAACTTTTCATCAGTTGC | PCR and Sequencing |
| 15E | AAAGACATACCAGACAGAGGG | AAACAGGACTTGTACTGTAGGA | PCR and Sequencing |
| 15F | CAGCCCCTTCAAGCAAACATG | TGGGAGTTTTCGCCATCCAC | PCR and Sequencing |
| 15G | TGTCTCTATCCACACATTCGTC | ATTTTCTTAGTTTCATTCTTCCTC | PCR and Sequencing |
| 15A-1 | GTTACTGCATACACATTGTGAC | GCTTTTTGTTTCCTAACATGAAG | Sequencing |
| 15A-2 | AGTACAAGGATGCCAATATTATG | ACTTCTATCTTTTTCAGAACGAG | Sequencing |
| 15A-3 | ATTTGAATACTACAGTGTTACCC | CTTGTATTCTAATTTGGCATAAGG | Sequencing |
| 15A-4 | CTGCCCATACACATTCAAACAC | TGTTTGGGTCTTGCCCATCTT | Sequencing |
| 15B-1 | AGTCCCATACACATTCAAACAC | GTTTCTCTTCATTATATTTTATGCTA | Sequencing |
| 15B-2 | AAGCCTACCAATTATAGTGAACG | AGCTGATGACAAAGATGATAATG | Sequencing |
| 15B-3 | AAGAAACAATACAGACTTATTGTG | ATGAGTGGGGTCTCCTGAAC | Sequencing |
| 15C-1 | ATCTCCCTCCAAAAGTGGTGC | TCCATCTGGAGTACTTTCTGTG | Sequencing |
| 15C-2 | AGTAAATGCTGCAGTTCAGAGG | CCGTGGCATATCATCCCCC | Sequencing |
| 15C-3 | CCCAGACTGCTTCAAAATTACC | GAGCCTCATCTGTACTTCTGC | Sequencing |
| 15D-1 | CCCTCCAAATGAGTTAGCTGC | TTGTGGTATAGGTTTTACTGGTG | Sequencing |
| 15D-2 | ACCCAACAAAAATCAGTTAGATG | GTGGCTGGTAACTTTAGCCTC | Sequencing |
| 15D-3 | ATGATGTTGACCTTTCCAGGG | ATTGTGTAACTTTTCATCAGTTGC | Sequencing |
| 15E-1 | AAAGACATACCAGACAGAGGG | CTTTTTTGGCATTGCGGAGCT | Sequencing |
| 15E-2 | AAGATGACCTGTTGCAGGAATG | GAATCAGACGAAGCTTGTCTAGTAT | Sequencing |
| 15E-3 | CCATAGTAAGTAGTTTACATCAAG | AAACAGGACTTGTACTGTAGGA | Sequencing |
| 15F-1 | CAGCCCCTTCAAGCAAACATG | GAGGACTTATTCCATTTCTACC | Sequencing |
| 15F-2 | TGGTTAATGGAGCCAATAAAAAGG | GTTGACTGGCGTACTAATACAG | Sequencing |
| 15F-3 | TGGTAATGGAGCCAATAAAAAGG | TGGGAGTTTTCGCCATCCAC | Sequencing |
| 15G-1 | TGTCTCTATCCACACATTCGTC | ATGTTTTTCATCCTCACTTTTTGC | Sequencing |
| 15G-2 | GGAGAAGAACTGGAAGTTCATC | TTGAATCTTTAATGTTTGGATTTGC | Sequencing |
| 15G-3 | TCTCCCACAGGTAATACTCCC | GCTAGAACTGAATGGGGTACG | Sequencing |
| 15G-4 | CAGGACAAAATAATCCTGTCCC | ATTTTCTTAGTTTCATTCTTCCTC | Sequencing |
