# Supplementary material for: APC Splicing Mutations Leading to In-Frame Exon 12 or Exon 13 Skipping Are Rare Events in FAP Pathogenesis and Define the Clinical Outcome
Source: Genes (Basel). 2021 Feb 28;12(3):353. doi: 10.3390/genes12030353 (PMC7997234; doi:10.3390/genes12030353)
Supplement: Supplementary file 1 [file genes-12-00353-s001.zip › genes-1080592/Supplementary_Table_3.docx]

| **Gene** | **Variant (Human Genome Variation Society, HGVS)** | **Chromosome position (GRCh37)** | **Location** | **Observed effect**  **on splicing** | **Effect on mRNA (HGVS)** | **Effect on protein (HGVS)** | **Clinical phenotype**  **(Classic FAP / AFAP)** | **Reference** |
| --- | --- | --- | --- | --- | --- | --- | --- | --- |
|  |  |  |  |  |  |  |  |  |
| ***APC*** | c.645+1G>T | chr5:g.112116601G>T | Intron 5 | Exon 5  skipping | r.532_645del | p.F178_Q215del | Classic FAP | [54] |
| ***APC*** | c.730-3C>G | chr5:g.112136973C>G | Intron 6 | Exon 7  skipping | r.730_834del | p.R244_Q278del | n.d. | [45] |
| ***APC*** | c.933G>C | chr5:g.112151290C>G | Exon 8 | Exon 8  skipping | r.835_933del | p.G279_K311del | n.d. | [67] |
| ***APC*** | c.1240C>T | chr5:g.112154969C>T | Exon 9 | Exon 9  partial skipping | r.934_1236del | p.V312_Q412del | n.d. | [67] |
| ***APC*** | c.1242C>T | chr5:g.112154971C>T | Exon 9 | Exon 9  partial skipping | r.934_1236del | p.V312_Q412del | n.d. | [67] |

Supplementary Table 3**.** APC splicing mutations leading to an in-frame protein and loss of APC regions not encompassing known functional sites/domains.

*n.d.: not discriminated*
